# COVID-19 and Tuberculosis Coinfection: An Overview of Case Reports/Case Series and Meta-Analysis

**DOI:** 10.3389/fmed.2021.657006

**Published:** 2021-08-24

**Authors:** Wan-mei Song, Jing-yu Zhao, Qian-yun Zhang, Si-qi Liu, Xue-han Zhu, Qi-qi An, Ting-ting Xu, Shi-jin Li, Jin-yue Liu, Ning-ning Tao, Yao Liu, Yi-fan Li, Huai-chen Li

**Affiliations:** ^1^Department of Respiratory and Critical Care Medicine, Shandong Provincial Hospital Affiliated to Shandong University, Jinan, China; ^2^Cheeloo College of Medicine, Shandong University, Jinan, China; ^3^Department of Geriatrics, People Hospital of Dongying District, Dongying, China; ^4^Department of Respiratory and Critical Care Medicine, Shandong Provincial Hospital Affiliated to Shandong First Medical University, Jinan, China; ^5^Department of Critical Care Medicine, Shandong Provincial Third Hospital, Jinan, China; ^6^Department of Respiratory and Critical Care Medicine, Beijing Hospital, Beijing, China; ^7^Graduate School of Peking Union Medical College, Chinese Academy of Medical Sciences and Peking Union Medical College, Beijing, China; ^8^First College of Clinical Medicine, Shandong University of Traditional Chinese Medicine, Jinan, China

**Keywords:** COVID-19, tuberculosis, co-infection, clinical features, risk factors

## Abstract

**Background:** Coronavirus disease 2019 (COVID-19) and tuberculosis (TB) are two major infectious diseases posing significant public health threats, and their coinfection (aptly abbreviated COVID-TB) makes the situation worse. This study aimed to investigate the clinical features and prognosis of COVID-TB cases.

**Methods:** The PubMed, Embase, Cochrane, CNKI, and Wanfang databases were searched for relevant studies published through December 18, 2020. An overview of COVID-TB case reports/case series was prepared that described their clinical characteristics and differences between survivors and deceased patients. Pooled odds ratios (ORs) with 95% confidence intervals (CIs) for death or severe COVID-19 were calculated. The quality of outcomes was assessed using GRADEpro.

**Results:** Thirty-six studies were included. Of 89 COVID-TB patients, 19 (23.46%) died, and 72 (80.90%) were male. The median age of non-survivors (53.95 ± 19.78 years) was greater than that of survivors (37.76 ± 15.54 years) (*p* < 0.001). Non-survivors were more likely to have hypertension (47.06 vs. 17.95%) or symptoms of dyspnea (72.73% vs. 30%) or bilateral lesions (73.68 vs. 47.14%), infiltrates (57.89 vs. 24.29%), tree in bud (10.53% vs. 0%), or a higher leucocyte count (12.9 [10.5–16.73] vs. 8.015 [4.8–8.97] × 10^9^/L) than survivors (*p* < 0.05). In terms of treatment, 88.52% received anti-TB therapy, 50.82% received antibiotics, 22.95% received antiviral therapy, 26.23% received hydroxychloroquine, and 11.48% received corticosteroids. The pooled ORs of death or severe disease in the COVID-TB group and the non-TB group were 2.21 (95% CI: 1.80, 2.70) and 2.77 (95% CI: 1.33, 5.74) (*P* < 0.01), respectively.

**Conclusion:** In summary, there appear to be some predictors of worse prognosis among COVID-TB cases. A moderate level of evidence suggests that COVID-TB patients are more likely to suffer severe disease or death than COVID-19 patients. Finally, routine screening for TB may be recommended among suspected or confirmed cases of COVID-19 in countries with high TB burden.

## Introduction

Coronavirus disease 2019 (COVID-19), caused by a novel beta-coronavirus, severe acute respiratory syndrome coronavirus 2 (SARS-CoV-2), has spread worldwide since December 2019, causing significant global public health and economic problems (1, 2). The World Health Organization declared COVID-19 a pandemic on March 11, 2020 (2). As of December 19, 2020, there have been over 75.7 million cases and 1.68 million deaths associated with COVID-19 worldwide (3). Nearly half of these cases involved four COVID-19 high-burden countries, including the United States (23.1%), India (13.2%), Brazil (9.5%), and Russia (3.7%) (3). Evidence to date suggests that COVID-19 patients with preexisting comorbidities such as hypertension, diabetes, and cardiovascular disease are at greater risk of death, but few studies have involved COVID-19 patients coinfected with other respiratory infectious diseases (4).

The initial signs and symptoms of COVID-19 are similar to other respiratory infections, such as tuberculosis (TB) and influenza. However, coinfections with common viral, bacterial, and fungal pathogens among COVID-19 patients are not unusual (5–7), which can interfere with the diagnosis and treatment of COVID-19. Before the COVID-19 outbreak, TB had been the most fatal infectious disease in the world for many years (8). Globally, an estimated 10 million people contracted TB and 1.4 million died from TB in 2019 (8). At present, evidence suggests that the main transmission route of both COVID-19 and TB is via respiratory droplets, and their main target are the lungs, which can lead to a worse outcome among COVID-19 and TB coinfection patients (aptly abbreviated COVID-TB) (7, 8). Therefore, due to the high prevalence of both of these infectious diseases and the potential worse prognosis of coinfection, an intensive investigation of COVID-TB cases may be of great clinical significance (3, 4, 8). However, few studies have focused on COVID-TB cases to date, and most of these are case reports involving only one patient, thus precluding systematic summaries of the clinical characteristics of coinfection cases (7, 9, 10). In addition, it is unclear whether COVID-TB patients have a worse prognosis or are more likely to develop severe disease, thus necessitating further study.

In this study, we aimed to more fully assess the impact of TB coinfection on COVID-19 patients using the following approach: (1) we present an overview of COVID-TB case reports or case series published through December 18, 2020 and describe the demographic characteristics, clinical symptoms, comorbidities, imaging features, laboratory indicators, type of TB coinfection, and treatment strategies for both COVID-TB survivors and non-survivors; (2) we performed a pooled analysis of published data regarding the odds ratios (ORs) of death or severe disease, comparing the COVID-TB and non-TB groups; and (3) we assessed the quality of outcomes using GRADEpro.

## Methods

### Search Strategy

An extensive search of the literature was conducted using the PubMed, Embase, Cochrane Central Register of Controlled Trials (CENTRAL), and two Chinese databases—the China National Knowledge Infrastructure (CNKI) and Wanfang databases—for articles published through December 18, 2020. The following keywords and Medical Subject Headings in partial or complete combinations were used in the search strategy of this review: “Coronavirus 2019,” “COVID-19,” “SARS CoV-2,” “COVID,” “novel coronavirus,” “2019-nCoV,” “severe acute respiratory syndrome,” “nCoV,” “CoV-2,” “SARS-2,” “new coronavirus,” and “tuberculosis.” The Chinese translations of these terms were searched in the CNKI and Wanfang databases (Table A1).

### Study Selection

We identified 6,919 publications, which were imported into EndNote X9 (Clarivate Analytics, Philadelphia, PA, USA), and 508 duplicate reports were excluded. First, three authors (WM. S., YF. L., and SJ. L.) reviewed the titles and abstracts for selection. Inclusion criteria were as follows: case reports or cases series of COVID-19 and TB coinfection; or original studies (retrospective or prospective clinical studies) that described the number/percentage of TB patients among confirmed COVID-19 cases. After preliminary screening, 149 full-text records were reviewed. Exclusion criteria were as follows: (1) case report or cases series without outcomes of COVID-TB cases (discharge or death); (2) original articles without the number/percentage of death/non-death or severe/non-severe cases among COVID-TB and COVID-19 subgroups; (3) sample size < 10 patients in the cohort study; and 4) publication overlap. The definition of severe COVID-19 is as follows: SpO_2_ <94% on room air at sea level; ratio of arterial partial pressure of oxygen to fraction of inspired oxygen (PaO_2_/FiO_2_) <300 mm Hg; respiratory frequency > 30 breaths/min; or lung infiltrates >50%.

### Data Extraction and Quality Evaluation

The following relevant data were extracted and collected in Excel: (1) baseline data, including the first author, country, publication date, type of study, number of patients with COVID-19, number of COVID-19 patients with TB, mean age, male/female ratio, outcome (survival or death), or clinical classification (severe or non-severe); (2) for case reports or cases series, we also collected detailed data on clinical symptoms, comorbidities, imaging features, laboratory indicators, and treatment strategies; and (3) for retrospective or prospective clinical studies, we collected the number/percentage regarding death/survival and severe/non-severe disease for the COVID-TB and COVID-19 subgroups.

The methodological quality of case reports or case series was evaluated using the Mayo Evidence-Based Practice Centre tool according to four domains (selection, ascertainment, causality, and reporting) (11). In addition, the methodological quality of other retrospective or cohort studies was assessed using the modified Newcastle–Ottawa scale (12). Two investigators (WM. S. and TT. X.) performed the analyses and summarized the scores of each study. Publications with a score ≥ 6 were considered of high quality and included in our analysis.

### Statistical Analysis

Eighty-nine COVID-TB cases were divided into survival and non-survival groups. Continuous variables including hematological and biochemical indicators of each group were described as the median (P_25_, P_75_) due to their skewed distribution. The Mann–Whitney *U* test was used for comparing two groups of continuous data (13). The mean and standard deviation of the age variable were described. Categorical variables, including age subgroup, sex, country, symptoms at admission, type of TB, computed tomography (CT) findings, and therapy, were expressed as frequencies and proportions. Categorical variables were compared using the chi-squared test or Fisher's exact test. Forest plots were prepared to show the pooled estimated ORs and associated 95% confidence intervals (CIs) for death from COVID-TB or severe disease, respectively. The heterogeneity of studies included in the Forest plots was assessed using Cochran's *Q* test and the *I*^2^ statistic. A fixed-effect model (inverse variance) was used when *I*^2^ was <50%. Otherwise, a random-effect model (DerSimonian–Laird) was used (14). A visual inspection of funnel plots was conducted to evaluate publication bias (Figures A1, A2), in which an asymmetric, inverted funnel shape usually indicates publication bias. Finally, we assessed the quality of outcomes using the GRADEpro software. A two-sided *p* < 0.05 was considered statistically significant. All statistical analyses were carried out using Review Manager (RevMan; version 5.3), SPSS (version 22.0), and GRADEpro (version 3.6.1).

## Results

### Characteristics of Included Studies

**Figure 1:**
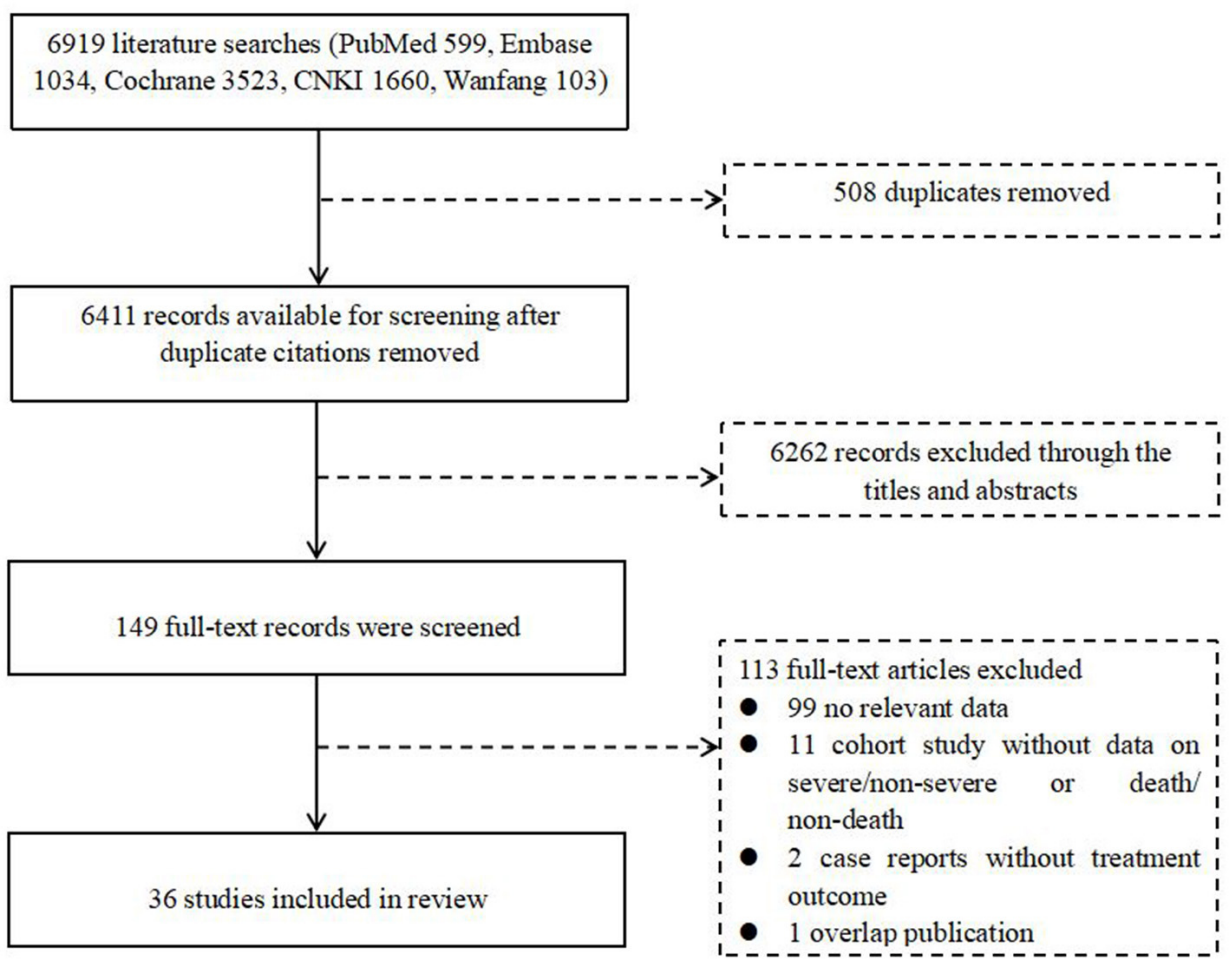
Flow diagram for the inclusion of studies. CNKI, Chinese National Knowledge Infrastructure.

A total of 6,919 articles were retrieved from the literature, including 599 from PubMed, 1,034 from Embase, 3,523 from Cochrane, 1,660 from CNKI, and 103 from Wanfang. After removing 508 duplicates, we evaluated the eligibility of 6,411 articles by screening the title and abstract, resulting in 149 studies being enrolled for full-text screening. Ultimately, we identified 36 eligible studies for our final analysis, of which 26 studies were used for an overview of COVID-TB cases, and 10 studies were used for the estimation of pooled ORs (Figure 1, Table 1).

**Table 1:** Summary of 36 studies included in the systematic review.

**References**	**Country**	**Publication time**	**Type of study**	**Number of patients with COVID-19**	**Number of COVID-TB patients**	**Mean age**	**Male/Female**	**Death or Severe**
Ata et al. (15)	India	2020 Aug	Case report	1	1	28	M	OD
Yousaf et al. (16)	Nepal/India/ Bangladesh	2020 Sep	Case series	6	6	35.5	6 M	OD
Yao et al. (17)	China	2020 Jul	Case series	3	3	50.33	3 M	1D
Vilbrun et al. (18)	Haiti	2020 Nov	Case report	1	1	26	M	OD
Tham et al. (19)	India/Bangladesh	2020 Jul	Case series	4	4	31.75	4 M	OD
Stochino et al. (20)	Italy	2020 Jul	Retrospective study	20	20	34.5	12 M/8 F	1D
Gupta et al. (21)	India	2020 Nov	Retrospective study	22	22	40.59	20 M/2 F	6D
Rivas et al. (22)	Panama	2020 Oct	Case series	2	2	41	2 M	OD
Luciani et al. (23)	Italy	2020 Oct	Case report	1	1	31	F	OD
Lopinto et al. (24)	France	2020 Sep	Case report	1	1	58	M	OD
Liu et al. (25)	China	2020 Jul	Case series	3	3	40	3 M	OD
He et al. (26)	China	2020 Oct	Case series	3	3	56.33	3 M	OD
Goussard et al. (27)	South Africa	2020 Sep	Case report	1	1	2	M	OD
Garg and Lee (28)	America	2020 Aug	Case report	1	1	44	M	OD
Gadelha Farias et al. (29)	Brazil	2020 Oct	Case series	2	2	41	2 M	OD
Freij et al. (30)	America	2020 Sep	Case report	1	1	5	F	1D
Faqihi et al. (31)	Saudi Arabia	2020 Jul	Case report	1	1	3	F	OD
Essajee et al. (32)	South Africa	2020 Sep	Case report	1	1	60	M	OD
Çinar et al. (33)	Turkey	2020 Oct	Case report	1	1	55	M	OD
Wang et al. (34)	China	2020 May	Case report	1	1	45	M	1D
Cao et al. (35)	China	2020 Sep	Case report	1	1	47	F	OD
Kumar et al. (36)	India	2020 Sep	Case report	1	1	38	M	1D
Motta et al. (37)	Italy/Spain	2020 May	Retrospective study	8	8	69.38	7 M/1 F	8D
Yadav and Rawal (38)	India	2020 Aug	Case report	1	1	43	M	OD
Sarma et al. (39)	India	2020 Nov	Case report	1	1	53	F	OD
Cao et al. (9)	China	2020 Oct	Case report	1	1	47	F	OD
Boulle et al. (40)	South Africa	2020 Aug	Population cohort study	22,308	2,128	——	15,256 M/7,052 F	625D (2.80%)
Liu et al. (41)	China	2020 Jul	Cohort study	1,190	24	57 (47, 67)	635 M/555 F	157D (13.19%)
Chen et al. (42)	China	2020 Sep	Retrospective study	55	1	74 (65–91)	34 M/22 F	19D (34.54%)
Du et al. (43)	China	2020 May	Prospective cohort study	179	8	57.6 ± 13.7	97 M/82 F	21D (11.73%)
Li et al. (44)	China	2020 Apr	Retrospective study	548	9	60 (48–69)	279 M/269 F	269S (49.09%)
Liu et al. (45)	China	2020 Jun	Retrospective study	342	2	56(45–67)	183 M/159 F	146S (42.69%)
Xiao et al. (46)	China	2020 Feb	Retrospective study	143	4	45.13 ± 1.04	73 M/70 F	36S (25.17%)
Zhang et al. (47)	China	2020 Apr	Retrospective study	1,350	5	44.1 ± 17.9	664 M/686 F	229S (16.96%)
Xu et al. (48)	China	2020 Apr	Retrospective study	23	2	46.0 (40.5, 52.0)	15 M/8 F	4S (17.39%)
Liu et al. (49)	China	2020 Mar	Retrospective study	36	13	47 ± 14	18 M/18 F	9S (25.00%)

*COVID-19, coronavirus disease 2019; COVID-TB, COVID-19 and tuberculosis coinfection*.

### Clinical Features of COVID-TB Cases

Table 2 describes the demographic characteristics, clinical symptoms, comorbidities, imaging features, laboratory indicators, treatment strategies, and type of TB among the overall group and the COVID-TB non-survivors and survivors. A total of 89 COVID-TB patients were included in the overview of case reports, of which 19 (23.46%) died, and 72 (80.9%) were male. The number and proportion of COVID-TB patients in the 0–14, 15–24, 25–44, 45–64, and 65+ years age groups were 4 (4.49%), 9 (10.11%), 40 (44.94%), 24 (26.97%), and 12 (13.48%), respectively, with an average age of 41.21 ± 17.84 years. The median age of the non-survivor group (53.95 ± 19.78 years) was older than that of the survivor group (37.76 ± 15.54 years) (*p* < 0.001). The non-survivors were less likely to be 25–44 years old compared with survivors (10.53 vs. 54.29%) but more likely than survivors to be in the 65+ years age group (47.37 vs. 4.29%) (*p* < 0.01). More than 85% of these cases were from India (*n* = 31, 4.83%), Italy (*n* = 26, 29.21%), and China (*n* = 12, 13.48%).

**Table 2:** Clinical characteristics of 89 patients with COVID-19 disease and tuberculosis.

**Clinical characteristics**	**All patients (*n =* 89)**	**Survivor (*n =* 70)**	**Non-survivor (*n =* 19)**	***p*-value**
**Age, years (** ***n =*** **89/70/19)**				
Average	41.21 ± 17.84	37.76 ± 15.54	53.95 ± 19.78	*P < * 0.001^***^
0–14	4 (4.49%)	3 (4.29%)	1 (5.26%)	1.000
15–24	9 (10.11%)	8 (11.43%)	1 (5.26%)	0.677
25–44	40 (44.94%)	38 (54.29%)	2 (10.53%)	0.001^**^
45–64	24 (26.97%)	18 (25.71%)	6 (31.58%)	0.609
65+	12 (13.48%)	3 (4.29%)	9 (47.37%)	*P < * 0.001^***^
**Sex (** ***n =*** **89/70/19)**				
Female	17 (19.1%)	13 (18.57%)	4 (21.05%)	0.809
Male	72 (80.9%)	57 (81.43%)	15 (78.95%)	0.809
**Country (** ***n =*** **89/70/19)**				
India	31 (34.83%)	24 (34.29%)	7 (36.84%)	0.836
Italy	26 (29.21%)	20 (28.57%)	6 (31.58%)	0.798
China	12 (13.48%)	10 (14.29%)	2 (10.53%)	1.000
Bangladesh	3 (3.37%)	3 (4.29%)	0 (0%)	1.000
Spain	3 (3.37%)	0 (0%)	3 (15.79%)	0.009^**^
Brazil	2 (2.25%)	2 (2.86%)	0 (0%)	1.000
South Africa	2 (2.25%)	2 (2.86%)	0 (0%)	1.000
America	2 (2.25%)	1 (1.43%)	1 (5.26%)	0.383
Panama	2 (2.25%)	2 (2.86%)	0 (0%)	1.000
Nepal	2 (2.25%)	2 (2.86%)	0 (0%)	1.000
Turkey	1 (1.12%)	1 (1.43%)	0 (0%)	1.000
France	1 (1.12%)	1 (1.43%)	0 (0%)	1.000
Haiti	1 (1.12%)	1 (1.43%)	0 (0%)	1.000
Saudi Arabia	1 (1.12%)	1 (1.43%)	0 (0%)	1.000
**TB (** ***n*** **= 89/70/19)**				
**Previous TB**	8 (8.99%)	7 (10.00%)	1 (5.26%)	1.000
**LTBI**	2 (2.25%)	2 (2.86%)	0 (0%)	1.000
**Site**				
Pulmonary TB only	71 (79.78%)	55 (78.57%)	16 (84.21%)	0.753
Extrapulmonary TB only	8 (8.99%)	5 (7.14%)	3 (15.79%)	0.360
Pulmonary TB/extrapulmonary TB (>1 site possible)	8 (8.99%)	8 (11.43%)	0 (0%)	0.194
**Site of extrapulmonary TB**				
Central nervous system	5 (5.62%)	3 (4.29%)	2 (10.53%)	0.289
Pleural	4 (4.49%)	4 (5.71%)	0 (0%)	0.574
lymphadenitis	2 (2.25%)	1 (1.43%)	1 (5.26%)	0.383
Gastrointestinal	1 (1.12%)	1 (1.43%)	0 (0%)	1.000
renal+ brain+ meningeal	2 (2.25%)	2 (2.86%)	0 (0%)	1.000
pericardial+ pleural+ splenic+ bone	1 (1.12%)	1 (1.43%)	0 (0%)	1.000
disseminated systemic tuberculosis	1 (1.12%)	1 (1.43%)	0 (0%)	1.000
**Comorbidities (** ***n =*** **78/61/17)**				
Any	44 (56.41%)	32 (52.46%)	12 (70.59%)	0.183
Hypertension	14 (17.95%)	6 (9.84%)	8 (47.06%)	*P < * 0.001^***^
Diabetes	19 (24.36%)	15 (24.59%)	4 (23.53%)	1.000
Hepatitis	3 (3.85%)	0 (0%)	3 (17.65%)	0.009^**^
Chronic kidney disease	3 (3.85%)	1 (1.64%)	2 (11.76%)	0.118
Cerebrovascular disease	2 (2.56%)	2 (3.28%)	0 (0%)	1.000
COPD	2 (2.56%)	1 (1.64%)	1 (5.88%)	0.391
Asthma	2 (2.56%)	2 (3.28%)	0 (0%)	1.000
Bronchiectasis	1 (1.28%)	1 (1.64%)	0 (0%)	1.000
Glioma	1 (1.28%)	1 (1.64%)	0 (0%)	1.000
Epilepsy	3 (3.85%)	3 (4.92%)	0 (0%)	1.000
HIV	5 (6.41%)	4 (6.56%)	1 (5.88%)	1.000
Cancer	2 (2.56%)	0 (0%)	2 (11.76%)	0.045^*^
Others	15 (19.23%)	11 (18.03%)	4 (23.53%)	0.729
**Symptoms at admission (** ***n =*** **81/70/11)**				
Fever	63 (77.78%)	52 (74.29%)	11 (100%)	0.111
Cough	52 (64.2%)	45 (64.29%)	7 (63.64%)	1.000
Dyspnea	29 (35.8%)	21 (30%)	8 (72.73%)	0.014^*^
Weight loss	13 (16.05%)	11 (15.71%)	2 (18.18%)	1.000
Fatigue	9 (11.11%)	8 (11.43%)	1 (9.09%)	1.000
Expectoration	8 (9.88%)	6 (8.57%)	2 (18.18%)	0.297
Chest pain	8 (9.88%)	8 (11.43%)	0 (0%)	0.590
Headache	7 (8.64%)	6 (8.57%)	1 (9.09%)	1.000
Myalgias	7 (8.64%)	7 (10%)	0 (0%)	0.585
Vomiting	6 (7.41%)	5 (7.14%)	1 (9.09%)	1.000
Chest tightness	2 (2.47%)	2 (2.86%)	0 (0%)	1.000
Diarrhea	2 (2.47%)	1 (1.43%)	1 (9.09%)	0.255
Reduced appetite	3 (3.7%)	3 (4.29%)	0 (0%)	1.000
Hemoptysis	4 (4.94%)	4 (5.71%)	0 (0%)	1.000
Sore throat	1 (1.23%)	0 (0%)	1 (9.09%)	0.136
Night sweats	2 (2.47%)	2 (2.86%)	0 (0%)	1.000
Chills	2 (2.47%)	2 (2.86%)	0 (0%)	1.000
Asymptomatic	4 (4.94%)	4 (5.71%)	0 (0%)	1.000
**CT findings (** ***n =*** **89/70/19)**				
Cavities	29 (32.58%)	23 (32.86%)	6 (31.58%)	0.916
Infiltrates	28 (31.46%)	17 (24.29%)	11 (57.89%)	0.005^**^
Ground-glass opacity	17 (19.1%)	15 (21.43%)	2 (10.53%)	0.347
Nodules	15 (16.85%)	14 (20%)	1 (5.26%)	0.177
Pleural effusion	10 (11.24%)	9 (12.86%)	1 (5.26%)	0.683
Fibrosis	11 (12.36%)	8 (11.43%)	3 (15.79%)	0.695
Patchy shadows	8 (8.99%)	8 (11.43%)	0 (0%)	1.000
Consolidation	8 (8.99%)	7 (10%)	1 (5.26%)	1.000
Miliary	5 (5.62%)	3 (4.29%)	2 (10.53%)	0.289
Reticules	5 (5.62%)	5 (7.14%)	0 (0%)	0.580
Calcific lesions	4 (4.49%)	4 (5.71%)	0 (0%)	0.574
Pleural thickening	3 (3.37%)	2 (2.86%)	1 (5.26%)	0.518
Lymphadenopathy	3 (3.37%)	3 (4.29%)	0 (0%)	1.000
Minimal signs of interstitial thickening	3 (3.37%)	2 (2.86%)	1 (5.26%)	0.518
Tree in bud	2 (2.25%)	0 (0%)	2 (10.53%)	0.044^*^
Air bronchogram	2 (2.25%)	2 (2.86%)	0 (0%)	1.000
Mediastinal emphysema	2 (2.25%)	1 (1.43%)	1 (5.26%)	0.383
Pleural empyema	2 (2.25%)	1 (1.43%)	1 (5.26%)	0.383
Atelectasis	2 (2.25%)	2 (2.86%)	0 (0%)	1.000
Fibrous stripes	1 (1.12%)	1 (1.43%)	0 (0%)	1.000
Bilateral	47 (52.81%)	33 (47.14%)	14 (73.68%)	0.040^*^
Unilateral	18 (20.22%)	17 (24.29%)	1 (5.26%)	0.105
**Therapy (** ***n =*** **61/51/10)**				
Antibiotics	31 (50.82%)	28 (54.9%)	4 (40%)	0.496
Anti-TB therapy	54 (88.52%)	45 (88.24%)	9 (90%)	1.000
Antiviral treatment	14 (22.95%)	13 (25.49%)	1 (10%)	0.429
Hydroxychloroquine	16 (26.23%)	14 (27.45%)	2 (20%)	1.000
Corticosteroids	7 (11.48%)	6 (11.76%)	1 (10%)	1.000
Traditional Chinese medicine	4 (6.56%)	4 (7.84%)	0 (0%)	1.000
Intravenous immunoglobulin	3 (4.92%)	3 (5.88%)	0 (0%)	1.000
Tocilizumab	2 (3.28%)	2 (3.92%)	0 (0%)	1.000
High-flow nasal cannula oxygen therapy	10 (16.39%)	8 (15.69%)	2 (20%)	0.663
Non-invasive mechanical ventilation	2 (3.28%)	2 (3.92%)	0 (0%)	1.000
ECMO	1 (1.64%)	1 (1.96%)	0 (0%)	1.000
Hemoperfusion	1 (1.64%)	1 (1.96%)	0 (0%)	1.000
Azithromycin	13 (21.31%)	12 (23.53%)	1 (10%)	0.674
Ceftriaxone	9 (14.75%)	9 (17.65%)	0 (0%)	0.332
Moxifloxacin	3 (4.92%)	2 (3.92%)	1 (10%)	0.421
Amikacin	2 (3.28%)	1 (1.96%)	1 (10%)	0.303
Meropenem	1 (1.64%)	1 (1.96%)	0 (0%)	1.000
Lopinavir/ritonavir	7 (11.48%)	7 (13.73%)	0 (0%)	0.587
Umifenovir hydrochloride	6 (9.84%)	6 (11.76%)	0 (0%)	0.577
Tenofovir	4 (6.56%)	4 (7.84%)	0 (0%)	1.000
Remdesivir	1 (1.64%)	0 (0%)	1 (10%)	0.164
Lamivudine	1 (1.64%)	1 (1.96%)	0 (0%)	1.000
Dolutegravir	1 (1.64%)	1 (1.96%)	0 (0%)	1.000
Favipiravir	1 (1.64%)	1 (1.96%)	0 (0%)	1.000
Interferon–α	5 (8.2%)	5 (9.8%)	0 (0%)	0.580
Low molecular weight heparin	2 (3.28%)	2 (3.92%)	0 (0%)	1.000
Aspirin	2 (3.28%)	1 (1.96%)	1 (10%)	0.303
**Laboratory examinations**				
Leucocyte count (reference range 3.5–9.5 × 10^9^/L) (*n =* 36/30/6)	8.25 (5.18–9.88)	8.015 (4.8–8.97)	12.9 (10.5–16.73)	0.007^**^
Neutrophil count (reference range 1.8–6.3 × 10^9^/L) (*n =* 14/13/1)	7.6 (6.93–7.6)	7.6 (6.77–7.6)	8.74	0.155
Lymphocyte count (reference range 1.1–3.2 × 10^9^/L) (*n =* 32/29/3)	0.99 (0.73–1.31)	1 (0.72–1.3)	0.9 (0.83–1.36)	0.721
Hemoglobin (reference range 115–150 g/L) (*n =* 16/14/2)	99 (83.5–114)	99 (85.5–120,75)	93.5 (88.25–98.75)	0.634
Platelet count (reference range 125–350 × 10^9^/L) (*n =* 7/4/3)	253 (201–323)	235.5 (209.5–259.75)	366 (273–398)	0.480
D-dimer (reference range 0–0.5 μg/mL) (*n =* 20/18/2)	1.41 (1.09–2.65)	1.41 (1.12–2.51)	3.11 (2.04–4.18)	0.801
CRP (reference range 0–8 mg/L) (*n =* 21/20/1)	77.1 (29.2–184.7)	67.05 (26.60–181.18)	293.8	0.137
ESR (reference range 0–20 mm/h) (*n =* 6/5/1)	75.5 (55.75–88.5)	70 (51–81)	123	0.143
PCT (reference range 0–0.05 ng/mL (*n =* 5/5/0)	2.57 (0.5–5.76)	2.57 (0.5–5.76)	—	—
FER (reference range 11.0–306.8 ng/mL) (*n =* 18/17/1)	739.5 (511.5–952.5)	768 (513–978)	137	0.102
ALT (reference range 13–35 U/L) (*n =* 14/13/1)	28.1 (25.05–36)	28.1 (28.1–33)	178	0.093
AST (reference range 7–40 U/L) (*n =* 3/3/0)	46 (27.55–78.5)	46 (27.55–78.5)	—	—
LDH (reference range 120–150 U/L) (*n =* 14/12/2)	384 (290.25–471.75)	350 (283–500.25)	433.5 (422.25–444.75)	0.465
Creatinine (reference range 62–106 umol/L) (*n =* 7/4/3)	335.92 (189.51–396.92)	189.51 (70.34–337.25)	335.92 (335.92–455.26)	0.154

*COVID-19, coronavirus disease 2019; TB, tuberculosis; LTBI, latent tuberculosis infection; COPD, chronic obstructive pulmonary disease; ECMO, extracorporeal membrane oxygenation; CRP, C-reactive protein; ESR, erythrocyte sedimentation rate; PCT, procalcitonin; FER, ferroprotein; ALT, alanine transaminase; AST, aspartate transaminase; LDH, lactate dehydrogenase;*

*** *p < 0.001*,

** *p < 0.01*,

* *p < 0.05*.

Among all 89 COVID-TB cases examined, 88.76% involved active TB, 8.99% had previous TB, and 2.25% had latent tuberculosis infection (LTBI). The proportions of pulmonary TB only, extrapulmonary TB only, and pulmonary TB/extrapulmonary TB (>1 site possible) were 79.78, 8.99, and 8.99%, respectively. Moreover, 5.62% were classified as central nervous system TB, 4.49% pleural TB, and 2.25% lymphadenitis. A total of 56.41% (44/78) of the COVID-TB patients had comorbidities, the most common of which was diabetes (24.36%, 19/78), followed by hypertension (17.95%, 14/78), HIV infection (6.41%, 5/78), hepatitis (3.85%, 3/78), epilepsy (3.85%, 3/78), chronic kidney disease (2.56%, 2/78), cerebrovascular disease (2.56%, 2/78), chronic obstructive pulmonary disease (2.56%, 2/78), asthma (2.56%, 2/78), or cancer (2.56%, 2/78). The non-survivors had more complications, such as hypertension (47.06 vs. 17.95%), hepatitis (17.65 vs. 0%), and cancer (11.76% vs. 0%), than survivors (*p* < 0.05). The 10 most common symptoms of COVID-TB at admission were fever (77.78%), cough (64.2%), dyspnea (35.8%), weight loss (16.05%), fatigue (11.11%), expectoration (9.88%), chest pain (9.88%), headache (8.64%), myalgia (8.64%), and vomiting (7.41%). The non-survivors had a higher percentage of dyspnea than survivors (72.73 vs. 30%) (*p* = 0.014).

In terms of treatment, 88.52% of the 61 COVID-TB patients received anti-TB therapy, 50.82% received antibiotics, 22.95% received antiviral therapy, 26.23% received hydroxychloroquine, 16.39% received oxygen therapy, 11.48% received corticosteroids, 8.2% received interferon-α, 6.56% received traditional Chinese medicine, and 4.92% received intravenous immunoglobulin. The most widely used antibiotic was azithromycin (21.31%), followed by ceftriaxone (14.75%), moxifloxacin (4.92%), amikacin (3.28%), and meropenem (1.64%). The antiviral drugs used included lopinavir/ritonavir (11.48%), umifenovir hydrochloride (9.84%), tenofovir (6.56%), remdesivir (1.64%), lamivudine (1.64%), dolutegravir (1.64%), and favipiravir (1.64%). These treatments did not differ significantly between survivors and non-survivors.

Features of lung imaging among the 89 patients were as follows: 52.81% had bilateral lesions, and 20.22% had unilateral lesions. The 10 most common imaging features included cavities (32.58%), infiltrates (31.46%), ground-glass opacity (19.1%), nodules (16.85%), pleural effusion (11.24%), fibrosis (12.36%), patchy shadows (8.99%), consolidation (8.99%), military lesions (5.62%), and reticules (5.62%). In addition, we found that non-survivors were more likely than survivors to have bilateral lesions (73.68 vs. 47.14%), infiltrates (57.89 vs. 24.29%), or tree in bud (10.53 vs. 0%) features (*p* < 0.05).

Elevated laboratory findings in COVID-TB patients included neutrophil count (7.60 [6.93–7.60] × 10^9^/L), D-dimer (1.407 [1.09–2.65] μg/ml), C-reactive protein (CRP, 77.10 [29.20–184.70] mg/L), erythrocyte sedimentation rate (ESR, 75.50 [55.75–88.50] mm/h), procalcitonin (PCT, 2.57 [0.50–5.76] ng/ml), ferroprotein (FER, 739.50 [511.50–952.50] ng/ml), aspartate transaminase (46 [27.55–78.50] U/L), lactate dehydrogenase (LDH, 384 [290.25–471.75] U/L), and creatinine (335.92 [189.51–396.92] μmol/L). Reduced laboratory indicators included lymphocyte count (0.99 [0.73–1.31] × 10^9^/L) and hemoglobin (99 [83.5–114] g/L). Leucocyte count, platelet count, and alanine transaminase levels were in the normal range. The nonsurvivors had a higher leucocyte count than the survivors (12.9 [10.5–16.73] vs. 8.015 [4.8–8.97] × 10^9^/L, *P* = 0.007). There were no significant differences between the survivors and non-survivors regarding the abovementioned laboratory examinations, except for the leucocyte count.

### Pooled ORs for Death or Severe COVID-TB vs. COVID-19

**Figure 2:**
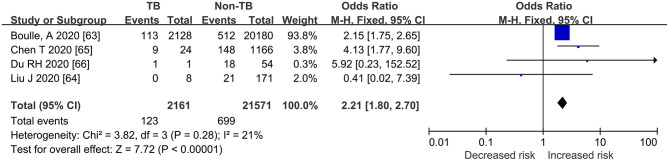
Forrest plot demonstrating the pooled ORs of death in COVID-19 patients with tuberculosis. ORs, odds ratios; TB, tuberculosis; CI, confidence interval; events refer to the occurrence of death.

**Figure 3:**
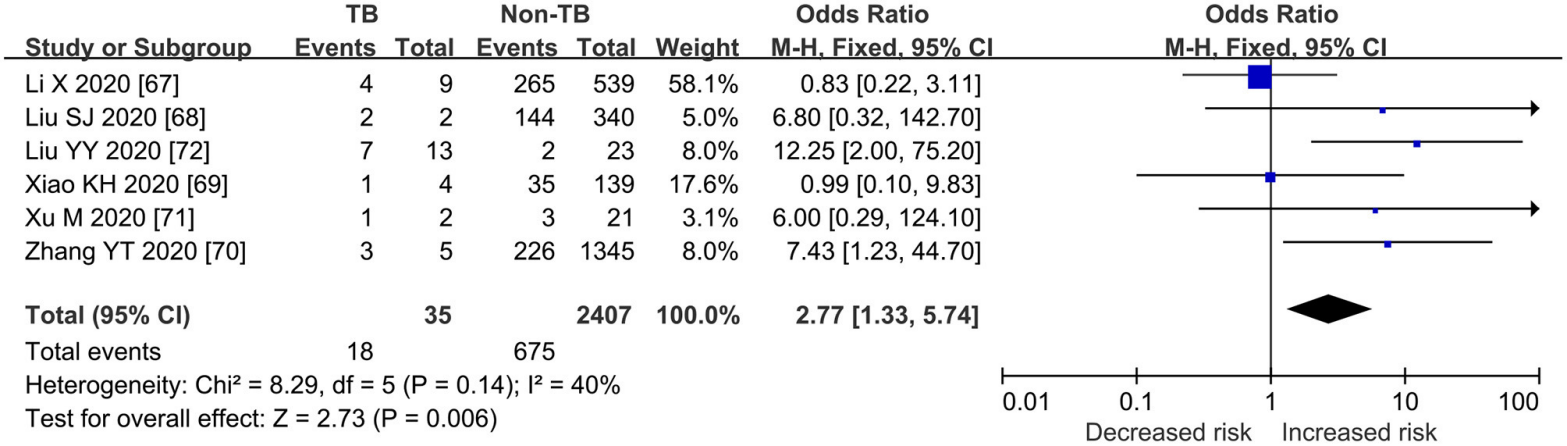
Forrest plot demonstrating the pooled ORs of severe cases in COVID-19 patients with tuberculosis. ORs, odds ratios; TB, tuberculosis; CI, confidence interval; events refer to the occurrence of severe cases.

As shown in Figures 2, 3, Table 3, the pooled ORs for death or severe disease in the COVID-TB group compared with the non-TB group were 2.21 (95% CI: 1.80, 2.70; heterogeneity: chi-squared = 3.82, df = 3, *p* = 0.28; *I*^2^ = 21%; Test for overall effect: *Z* = 7.72, *p* < 0.00001) and 2.77 (95% CI: 1.33, 5.74; heterogeneity: chi-squared = 8.29, df = 5, *p* = 0.14; *I*^2^ = 40%; Test for overall effect: *Z* = 2.73, *p* < 0.006), with a moderate level of evidence. The percentages of deaths and cases of severe disease, respectively, were 5.69% (123/2,161) and 51.43% (18/35) in the COVID-TB group, 3.24% (699/21,571) and 28.04% (675/2,407) in the non-TB group, and 3.46% (822/23,732) and 28.38% (693/2,442) in the overall group. The proportion of TB among the non-survivor, survivor, and overall group was 14.96% (123/822), 8.90% (2038/22,910), and 9.11% (2,161/23,732), respectively. Moreover, the proportion of TB among the severe, non-severe, and overall patient groups was 2.60% (18/693), 0.97% (17/1,749), and 1.43% (35/2,442), respectively.

**Table 3:** GRADEpro assessment of methodologic quality of included studies examining the ability of TB to increase the risk of death or severe cases among COVID-19.

**Risk of death or severe cases among COVID-TB compared to COVID-19** **Patient or population: [COVID-19]** **Settings:** **Intervention: TB** **Comparison: non-TB**						
**Outcomes**	Illustrative comparative risks^*^ (95% CI)		**Relative effect** **(95% CI)**	**No of Participants (studies)**	**Quality of the evidence (GRADE)**	**Comments**
	**Assumed risk**	**Corresponding risk**				
	**Non-TB**	**TB**				
Death	Study population		**OR 2.21** (1.8 to 2.7)	23,732 (4 studies)	⊕⊕⊕⊖ **moderate**^a^	
	32 per 1,000	69 per 1,000 (57 to 83)				
	Moderate					
	125 per 1,000	240 per 1,000 (205 to 278)				
Severe	Study population		**OR 2.77** (1.33 to 5.74)	2,442 (6 studies)	⊕⊕⊕⊖ **moderate**^a^	
	280 per 1,000	519 per 1,000 (341 to 691)				
	Moderate					
	210 per 1,000	424 per 1,000 (261 to 604)				

* *The basis for the **assumed risk** (e.g., the median control group risk across studies) is provided in footnotes. The **corresponding risk** (and its 95% confidence interval) is based on the assumed risk in the comparison group and the **relative effect** of the intervention (and its 95% CI). **CI**, Confidence interval; **OR:** Odds ratio*.

*GRADE Working Group grades of evidence **High quality:** Further research is very unlikely to change our confidence in the estimate of effect. **Moderate quality:** Further research is likely to have an important impact on our confidence in the estimate of effect and may change the estimate. **Low quality:** Further research is very likely to have an important impact on our confidence in the estimate of effect and is likely to change the estimate*.

***Very low quality:** We are very uncertain about the estimate*.

a *Relative risk > 2*.

*COVID-19, coronavirus disease 2019; TB, tuberculosis. Bold indicates the pooled ORs of for death or severe disease in the cases in COVID-TB group compared with the non-TB group were as 2.21 and 2.77 respectively, with a moderate level of evidence*.

## Discussion

This meta-analysis of 36 studies provided a summary of demographic, radiological, and laboratory characteristics as well as symptoms at admission, comorbidities, therapy, and outcomes of COVID-TB cases and investigated the impact of TB on the prognosis of COVID-19 patients. Our study showed that non-survivors were older and had more complications associated with hypertension, hepatitis, and cancer, had more symptoms of dyspnea, and were more likely to have CT imaging features of bilateral lesions, infiltrates, tree in bud, and higher leucocyte count than survivors. We also found a moderate level of evidence indicating that COVID-TB patients are at higher risk of death or serious illness than COVID-19 patients without TB.

Older age, especially >65 years, may be a risk factor for death from COVID-TB, consistent with previous findings indicating that the mortality rate from COVID-19 increases exponentially with age (50, 51). According to a model-based analysis, the estimated overall death rate for COVID-19 was 0.66%, but increasing to 7.8% among patients aged >80 years and decreasing to 0.0016% among children aged <9 years (52). There are several primary reasons for these differences, including more preexisting comorbidities, dysregulation in the immune response, and chronic subclinical systemic inflammation (inflammaging) among older adults than younger persons (53). Thus, the elderly should be the primary focus of both COVID-19 and COVID-TB mitigation efforts due to its much higher mortality risk in that group.

COVID-TB patients had a much higher rate of comorbidities than COVID-19 patients (56.41 vs. 25.1%) (54). The most prevalent comorbidities among COVID-19 patients were hypertension (21.1, 95% CI: 13.0–27.2%), diabetes (9.7%, 95 CI: 7.2–12.2%), cardiovascular disease (8.4%, 95% CI: 3.8–13.8%), and respiratory system disease (1.5%, 95% CI: 0.9–2.1%), whereas the most common comorbidities among COVID-TB patients were diabetes (24.36%), hypertension (17.95%), HIV infection (6.41%), hepatitis (3.85%), epilepsy (3.85%), and cancer (2.56%) (54). Indeed, both HIV infection and diabetes are important risk factors for TB infection (55). Interestingly, we found that COVID-TB patients who died had a much higher proportion of hypertension (47.06 vs. 9.84%) and cancer (11.76 vs. 0%) than those who survived. A previous study also indicated that underlying diseases such as hypertension (OR = 2.72, 95% CI: 1.60, 4.64) and diabetes (OR = 3.68, 95% CI: 2.68, 5.03) are risk factors for critical disease/mortality (56). Early publications reported a “harmful hypothesis” that SARS-CoV-2 binds to target cells via angiotensin-converting enzyme 2 (ACE2), and patients with hypertension usually have increased expression of ACE2 due to the use of renin angiotensin system inhibitors (57). Although some studies indicated that ACE inhibitors (angiotensin converting enzyme inhibitors) and ARB (angiotensin-receptor blockers) therapy was harmful in COVID-19 patients, an updated meta-analysis concluded that ACEI/ARB therapy does not contribute to increased risk of mortality or severe manifestations among COVID-19 patients (58, 59). It was recommended that ACEI/ARB therapy be continued among patients with coexisting hypertension (60). However, whether the guidelines regarding ACEI/ARB therapy among COVID-19 patients are equally applicable to COVID-TB patients remains to be determined.

The most common clinical manifestations of COVID-TB are fever, cough, dyspnea, weight loss, fatigue, and expectoration (13, 61). Existing evidence indicates that the features of lung imaging among COVID-19 patients include bilateral involvement, peripheral distribution, mixed ground-glass opacity and consolidation, and vascular thickening (62), whereas the most common CT findings of COVID-TB include bilateral lesions, cavities, infiltrates, ground-glass opacity, nodules, pleural effusion, and fibrosis. Thus, clinicians should take COVID-TB coinfection into consideration upon encountering the above CT imaging features in the future instead of just focusing on one disease. The increased prevalence of dyspnea and CT findings including bilateral lesions, infiltrates, and tree in bud among COVID-TB patients who died suggests that they may be good predictors of disease severity, in line with the findings of previous studies (13, 61).

Markedly elevated levels of inflammatory markers, including CRP, ESR, PCT, FER, and LDH, slightly increased neutrophil count and D-dimer level, and decreased lymphocyte count and hemoglobin level were observed in COVID-TB patients. However, we did not find any significant differences in these indexes (except the leucocyte count) between the survivors and non-survivors, which was inconsistent with previous findings that inflammatory markers were elevated in severe disease and critically ill groups (63, 64). The findings regarding the characteristics of COVID-TB biomarkers may provide references for conventional hematological and inflammatory examinations for disease severity classification, and early warning of progression (65).

According to the COVID-19 treatment guidelines, the main treatments include antiviral therapy, immune-based therapy, and adjunctive therapy (66). Antiviral therapies may have a greater effect in the early course of COVID-19, whereas immunosuppressive/anti-inflammatory therapies will be more beneficial in the later stages (66, 67). Although previous studies indicated that corticosteroids are associated with a reduction in short-term mortality and the need for mechanical ventilation in COVID-19 patients, whether immunosuppressive therapies such as dexamethasone, a corticosteroid, can be used in COVID-TB patients as well has not been investigated (68). In our study, although there was no statistically significant difference in the proportion of corticosteroid therapy among COVID-TB survivors and non-survivors, we still recommend a more cautious use of corticosteroids in COVID-TB patients because of the potential increased risk of active or severe TB infection associated with corticosteroid use. Studies involving larger samples are needed to explore the impact of corticosteroid therapy on the prognosis of COVID-TB patients (69). It is also worthwhile to explore whether COVID-19 patients with active TB, LTBI, or previous TB should receive standard anti-TB treatment.

Based on this meta-analysis, we found that COVID-TB patients were 2.21 and 2.27 times more likely to die or develop severe COVID-19, respectively. In many countries, the ongoing COVID-19 pandemic coincides with other major public health problems, especially TB, and the impact of the COVID-19 pandemic may be ameliorated if we continue to implement health-care services and key prevention measures (70, 71). COVID-TB infection is a novel disease that remains to be further explored and needs more attention in high-TB burden countries such as India, Indonesia, and China (8). Encouragingly, it has been reported that use of the GeneXpert MTB/RIF platform for the surveillance of COVID-19 is relevant and achievable, especially in low-income and middle-income countries without sufficient classical real-time PCR capabilities but with an already existing GeneXpert MTB/RIF network (72).

Our study has some strengths. First, detailed information was collected in our study, including data regarding demographic characteristics, imaging findings, symptoms at admission, comorbidities, therapies, and outcomes, and the synthesis of these characteristics may provide further guidance for clinicians in terms of diagnosis and treatment of COVID-TB. Second, a comprehensive literature search of both Chinese and English language databases were performed, resulting in a more accurate evaluation of summary estimates with higher precision. Third, the studies included in our meta-analysis had relatively low levels of heterogeneity.

Our study also has several limitations. First, although we performed an extensive search of the literature, most of the eligible studies included in the Forest plots were Chinese. Second, some detailed patient information was not available due to publication bias or no relevant laboratory tests having been performed. Finally, the sample size of our case overview was still limited; thus, further large cohort studies of COVID-TB are needed.

## Conclusion

In summary, older age, complications including hypertension, hepatitis, cancer, symptoms of dyspnea at admission, CT imaging features of bilateral lesions, infiltrates, tree in bud, and higher leucocyte count may be predictors for poor prognosis of COVID-TB patients. Furthermore, a moderate level of evidence suggests that people with COVID-TB are 2.21 and 2.27 times more likely to die or develop severe disease, respectively, than COVID-19 patients. Finally, routine screening for *Mycobacterium tuberculosis* is recommended among suspected or confirmed cases of COVID-19 in high–TB burden countries due to the worse prognosis of COVID-TB and the confounding clinical symptoms of these two diseases.

## Data Availability Statement

The raw data supporting the conclusions of this article will be made available by the authors, without undue reservation.

## Author Contributions

H-cL, W-mS, and Y-fL conceived and designed the study. H-cL, X-hZ, Q-qA, S-qL, and YL directed its implementation including the data analysis and writing of the paper. W-mS and Y-fL analyzed the data. W-mS, YL, Q-yZ, J-yL, T-tX, S-jL, X-hZ, and N-nT contributed to data collection, materials, and analytic tools. W-mS and H-cL wrote and revised the manuscript. All authors revised it critically for important intellectual content, gave final approval of the version to be published and agreed to be accountable for all aspects of the work in ensuring that questions related to the accuracy or integrity of any part of the work were appropriately investigated and resolved.

## Conflict of Interest

The authors declare that the research was conducted in the absence of any commercial or financial relationships that could be construed as a potential conflict of interest.

## Publisher's Note

All claims expressed in this article are solely those of the authors and do not necessarily represent those of their affiliated organizations, or those of the publisher, the editors and the reviewers. Any product that may be evaluated in this article, or claim that may be made by its manufacturer, is not guaranteed or endorsed by the publisher.

## Supplementary Material

Table A1 Search strategies for PubMed, Embase, Cochrane, CNKI, and Wanfang. CNKI, Chinese National Knowledge Infrastructure.

Figure A1 Funnel plot to detect publication bias.

Figure A2 Funnel plot to detect publication bias.
