# Supplementary material for: COVID-19 and Tuberculosis Coinfection: An Overview of Case Reports/Case Series and Meta-Analysis
Source: Front Med (Lausanne). 2021 Aug 24;8:657006. doi: 10.3389/fmed.2021.657006 (PMC8421570; doi:10.3389/fmed.2021.657006)
Supplement: Table A1 — Search strategies for PubMed, Embase, Cochrane, CNKI, and Wanfang. CNKI, Chinese National Knowledge Infrastructure. [file Table_1.doc]

| **Database** | **Search** | **Results** | **Time** |
| --- | --- | --- | --- |
| **PubMed** | (((((((((((COVID) OR (COVID-19)) OR (novel coronavirus)) OR (new coronavirus)) OR (coronavirus 2019)) OR (2019-nCoV)) OR (nCoV)) OR (CoV-2)) OR (SARS-2)) OR (SARS CoV-2)) OR (severe acute respiratory syndrome coronavirus 2)) AND (tuberculosis) | [599](https://pubmed.ncbi.nlm.nih.gov/?term=(((((((((((COVID)+OR+(COVID-19))+OR+(novel+coronavirus))+OR+(new+coronavirus))+OR+(coronavirus+2019))+OR+(2019-nCoV))+OR+(nCoV))+OR+(CoV-2))+OR+(SARS-2))+OR+(SARS+CoV-2))+OR+(severe+acute+respiratory+syndrome+coronavirus+2))+AND+(tuberculosis)&sort=) | 2020/12/18 |
| **Embase** | (covid OR 'covid 19' OR (novel AND coronavirus) OR (new AND coronavirus) OR (coronavirus AND 2019) OR '2019 ncov' OR ncov OR 'cov 2' OR 'sars 2' OR (sars AND 'cov 2') OR (severe AND acute AND respiratory AND syndrome AND coronavirus AND 2)) AND tuberculosis | 1034 | 2020/12/18 |
| **Cochrane** | COVID in All Text OR COVID-19 in All Text OR coronavirus in All Text OR SARS-2 in All Text AND tuberculosis in All Text - with Cochrane Library publication date in The last year (Word variations have been searched) | 3523 | 2020/12/18 |
| **CNKI** | （full text：新型冠状病毒）OR（full text：新冠肺炎）OR（full text：冠状病毒病）OR（full text：2019-nCoV）OR（full text：COVID-19）AND（full text：结核） | 1660 | 2020/12/18 |
| **Wanfang** | ((((新冠肺炎) OR 冠状病毒) OR 2019-nCoV) OR COVID-19) AND 结核 | 103 | 2020/12/18 |

**Appendix Table 1** Search strategies for PubMed, Embase, Cochrane, CNKI, and Wanfang

**Notes:** CNKI, Chinese National Knowledge Infrastructure
