# Supplementary figures and images for: COVID-19 and Tuberculosis Coinfection: An Overview of Case Reports/Case Series and Meta-Analysis
Source: Front Med (Lausanne). 2021 Aug 24;8:657006. doi: 10.3389/fmed.2021.657006 (PMC8421570; doi:10.3389/fmed.2021.657006)

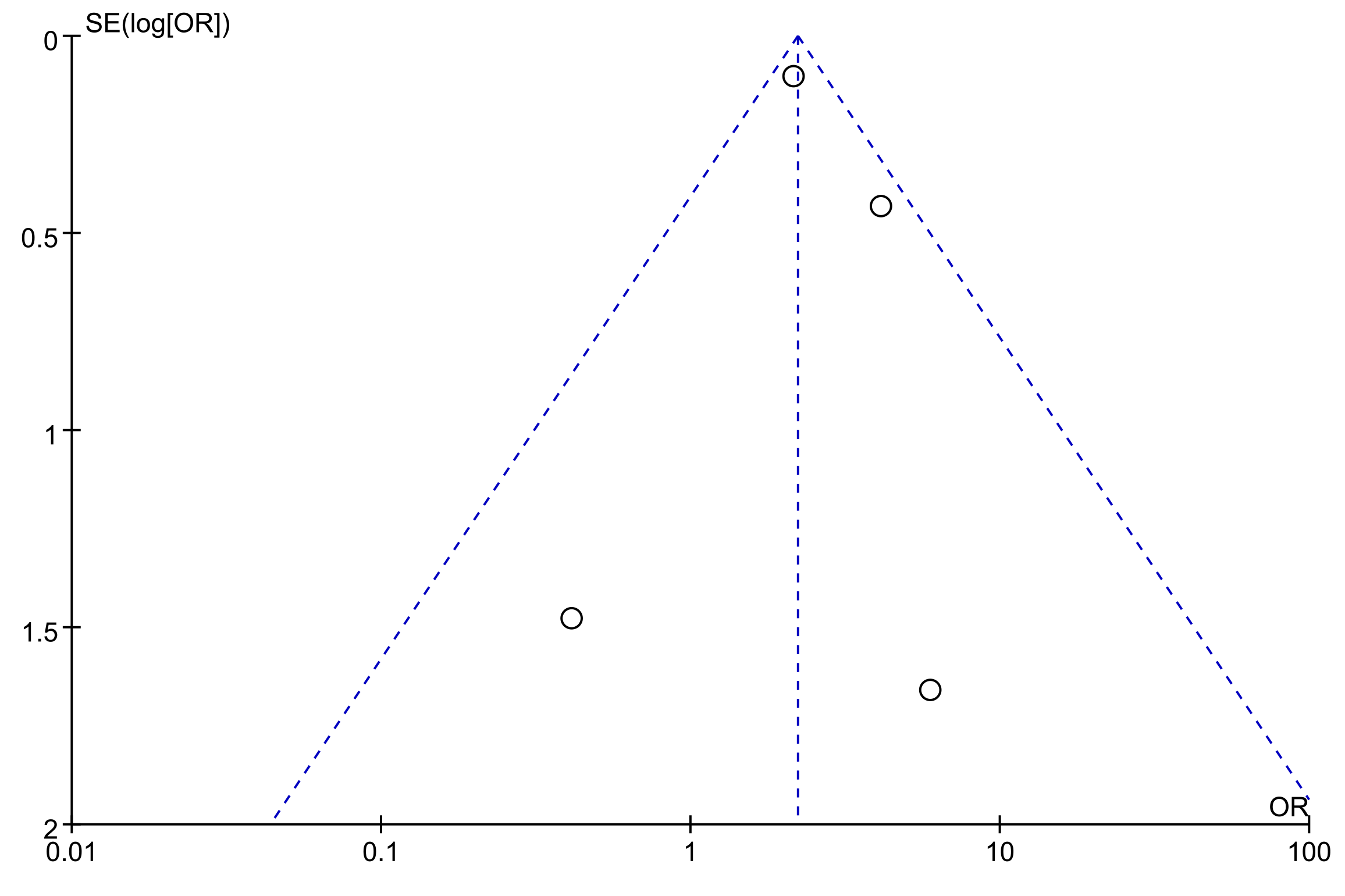

Supplement: Figure A1 — Funnel plot to detect publication bias. [file Image_1.TIF]

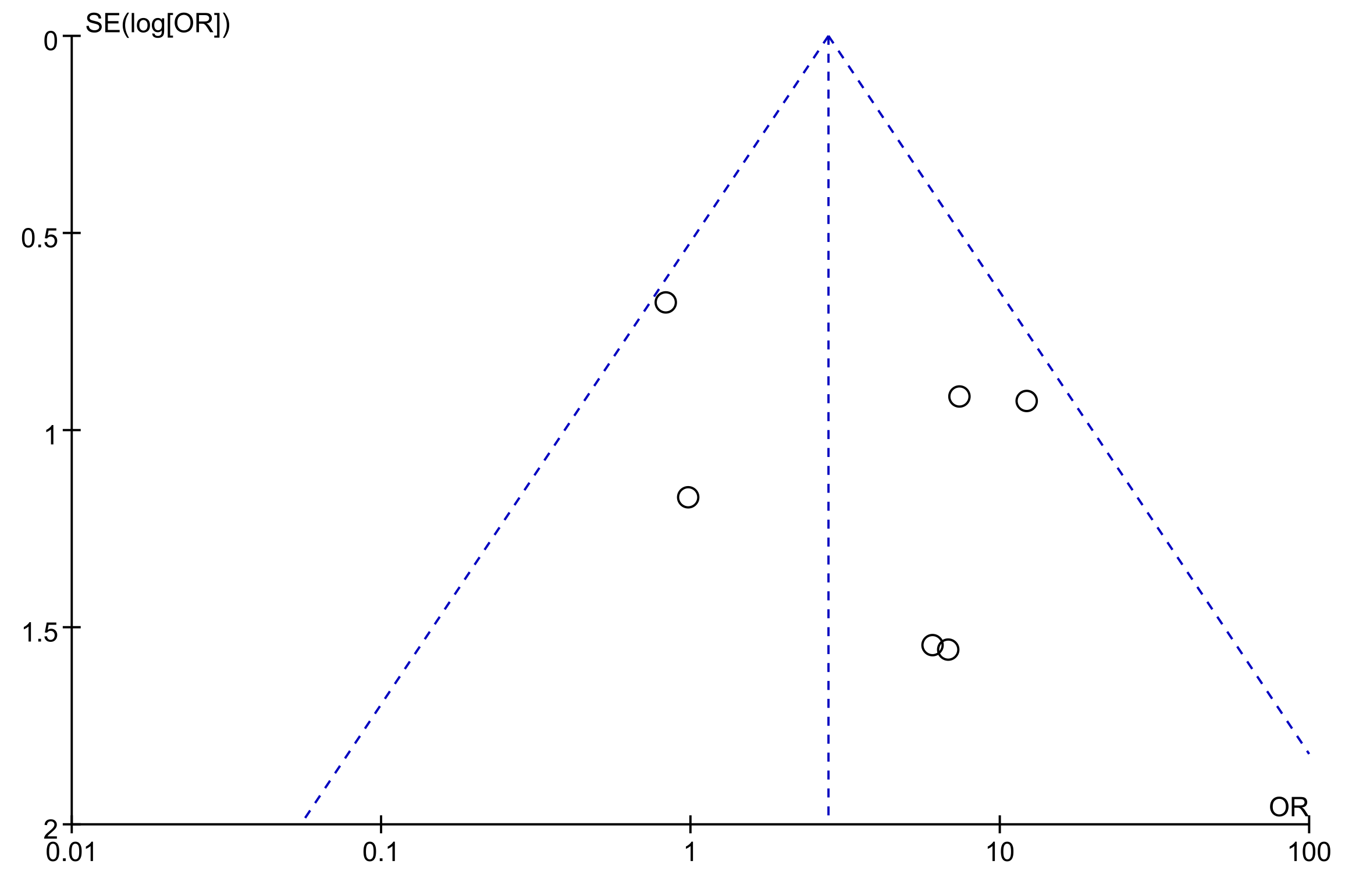

Supplement: Figure A2 — Funnel plot to detect publication bias. [file Image_2.TIF]
